# Editorial Correspondence

**Published:** 1851-07

**Authors:** 


### EDITORIAL CORRESPONDENCE.

Dr. Taylor, Sir :—Permit me through the medium of the Register, to reply to some of the numerous inquiries that have been made by correspondents within the last few weeks, with reference to my new method of setting artificial teeth upon metalic plates, which consists in flowing in an artificial gum, between, and upon the teeth, and uniting them firmly to each other, and to the plate upon which they are set, by means of a fusible silicious cement, which was noticed in the last number of the Register.

Dr. B. desires to know whether the method of setting teeth can be employed upon ordinary gold plate. *Answer:* not with perfect safety, for the degree of heat necessary to fuse the cement would probably melt the plate, especially if it be not more than sixteen or eighteen carets fine. I use gold twenty-two carets with no alloy but platina. I also use platina plates which I think still better, as there is less contraction or expansion in platina than gold, when subjected to a strong heat.

He wishes to know whether the teeth can be be kept in their proper position upon the plate while the gum is being flowed upon them.

*Answer:* by this method, the teeth maintain precisely the position in which they are placed upon the plate. This is done by means of a composition, with which the teeth are covered preparatory to fusing the cement, which neither contracts, expands, nor cracks, when subjected to a white heat, consequently no tooth in a full upper or lower set, can become displaced in the least by the fusion of the cement.

The composition has been sought out for this purpose by the inventor of this method of setting teeth, as there are no other substances now known in use, which possess the requisite properties.

S. thinks it may do, if there is any way to repair them when they get broken off the plate. It seems hardly necessary to provide for a contingency which is not likely to occur, for no ordinary use in the mouth can break them. But if by a fall, or otherwise, a tooth should get broken, it can be readily replaced with a new one, by means of the cement, and so perfect in appearance as to elude detection, and with much greater facility, and less trouble than to solder on a tooth in the usual way.

W. is of the opinion that the teeth would be still more secure upon the plate if they were soldered on. From experience thus far, we find the cement amply sufficient, without any other fastenings. But where it is desired, back plates, and also clasps for partial sets of teeth, can be soldered on with great facility, and that too without the use of the blow-pipe or soldering lamp.

In short, this method as a whole, (for it embraces a number of new and important features,) constitutes an entire new system, which presents to the public the dawn of a new era in the history of the dental art, for by this means the profession will be enabled to reach a higher degree of perfection in the artificial department, than it has ever before attained, and in point of BEAUTY, STRENGTH, CLEANLINESS, DURIBILITY, ECONOMY, FACILITY and SIMPLICITY, it cannot be surpassed. Numerous persons desire to know in what way the profession is to acquire a knowledge of it. *Answer;* by written or oral and practical instruction—the latter is preferable. This will necessarily involve some expence. The inventor has devoted HIMSELF closely to this subject between eight and nine years, at an enormous expenditure, for the purpose of developing the principles and gaining the points he has at length attained, believing that a generous public will cheerfully reciprocate in its response for that which tends to promote the general good, and that a liberal profession will be ready and willing to reimburse a member through whose instrumentality it has been brought forward to a stand point never before occupied.

The inventor would prefer giving practical instructions at his own laboratory, to those who are within a convenient distance, and who desire to acquire a knowledge of this method of setting teeth.

Those who reside at a distance can be furnished with written instructions published in pamphlet form.

The times of imparting instruction must necessarily vary according to circumstances, but a practical dentist can acquire the modus operandi in from one to two days. Those who are students in the Ohio Dental College, will possess advantages in this respect that others cannot reasonably expect, as the inventor occupies a chair in that institution.

— J. ALLEN.

We give place to Dr. Allen's letter, merely having room to say that we regard the color of the gum which the Dr. uses as much improved since we first noticed it. We believe the Dr. has spent much time and money in perfecting this improvement, for we regard it in *very many cases* as a great improvement, which must lead to a great change in mechanical dentistry. The quality of the materials used must necessarily be far better than is generally used by the profession, and this we think is of some consequence. The Dr. regarding it strictly as an improvement in mechanical dentistry, has, we believe, taken steps to obtain a patent. To this we are aware many of the profession will object, at present we would merely remark that we believe that Dr. Allen will give the profession no just cause of complaint.

